# Music, drama, and social development in Portuguese children

**DOI:** 10.3389/fpsyg.2023.1093832

**Published:** 2023-06-01

**Authors:** Graça Boal-Palheiros, Beatriz Ilari

**Affiliations:** ^1^CIPEM/INET-md, Escola Superior de Educação, Politécnico do Porto, Porto, Portugal; ^2^Department of Music Teaching and Learning, University of Southern California, Los Angeles, CA, United States

**Keywords:** social competence, social skills, school-aged children, music education, drama education, primary school, disadvantaged backgrounds

## Abstract

**Introduction:**

Social competence plays a fundamental role in children’s development, and in their functioning at school and in life. Social skills, as learned behaviors that allow children to positively interact with others, are important for success in both academic and peer-group settings. Children’s participation in collective music and other arts education has been associated with the development of social skills. However, different measures and diverse programs adopted in various studies make it difficult to contrast study findings. Additionally, research with children from low-income families remains scarce. The aim of this study was to examine the role of music and drama education programs in primary schools on the development of social skills of Portuguese children from disadvantaged communities. Both programs were carefully designed with performing, creating, and listening activities, and were delivered by specialist and experienced teachers/performers, who used active and participatory teaching strategies.

**Methods:**

In our longitudinal design with pre- and post-evaluations, we used the Social Skills Rating System or SSRS—Teacher Form, adapted for the Portuguese population. Classroom teachers rated their students’ social skills on a 3-point scale in three domains: Social skills (Cooperation, Assertion, Self-control), Behavioral problems (Externalizing problems, Internalizing problems, Hyperactivity) and, on a 5-point scale, Academic competence.

**Results and Discussion:**

Our findings suggest that participation in music and drama programs during one school year improved children’s assertion and self-control, and cooperation in the drama group. Participation in the music and drama programs also appeared to serve as a protective factor by reducing externalizing, internalizing and behavioral problems. These findings are discussed in light of previous studies along with limitations and directions for future research.

## 1. Introduction

Social competence is a vital resilience factor that is known to increase positive developmental outcomes in the face of adversity (Milligan et al., 2017). Socially competent children are usually able to form and sustain friendships and develop positive relationships with peers and adults, which may lead to peer collaboration, enhanced problem-solving skills and academic achievement in school-aged children (Milligan et al., 2017). Social competence has also been described as a protective factor for mental health, and as a predictor for later career success. While there is a general agreement about the central role of social competence in children’s development, definitions of social competence vary considerably (Milligan et al., 2017). Central to many definitions of this complex construct is the notion that social competence relies on the use of social skills “in a way that adheres to social conventions and that responds appropriately to others’ emotions and thoughts” (Milligan et al., 2017, p. 64). That is, definitions of social competence usually encompass intra and interpersonal skills that can be grouped into overt components such as cooperation, assertion, empathy, self-control, and responsibility (Sørlie et al., 2021). Thus, social competence is multifaceted and involves social, emotional, behavioral, and cognitive components that enable social adaptation.

Social skills are observable, behavioral components of social competence (Little et al., 2017; Sørlie et al., 2021), and arguably the “most malleable” (Little et al., 2017). Erath (2009) defines social skills in childhood as “behaviors that enable children to elicit positive responses from others and establish positive relationships with others” (p. 1563). The development and acquisition of social skills during childhood is critical, not only given its links to children’s interactions with peers and family members, but also due to their associations with social adjustment over time and across multiple life domains. Social skills are learned in, modeled, and affected by the contexts of children’s daily lives. Children develop social skills over time, through interpersonal interactions and experiences at home, school, and community settings. Schools play a central role in the development of children’s social skills. Children typically spend a considerable amount of time in schools, where they have ample opportunities to exercise their social skills in dyads, small and large groups, while interacting with peers, teachers, and staff (Sørlie et al., 2021). The development of social skills is also linked to age and maturation. At least on a theoretical level, there is reason to expect children who score high on measures of social skills at one point in time to continue scoring high at a later time, and to also expect an increase in social skills scores during the years of primary school (Sørlie et al., 2021).

Whereas social skills are important in their own right, they are also related to other areas including mental health and academic achievement (Sørlie et al., 2021). In recent years, educators, scholars, and policy makers have focused on socio-emotional learning (SEL), not just social skills or social competence. Several reports position socio-emotional skills as central elements of success, in school and in life (see Holochwost et al., 2021). For example, the OECD’s Survey on Social and Emotional Skills reports data from a large international sample of students, parents, and teachers on the skills of 10- and 15-year-old learners in nine countries across the world. The results of this survey confirm that social and emotional skills matter for academic achievement and well-being, and that they differ by gender, social background, and age. Additionally, students from affluent backgrounds reported higher socio-emotional skills than their disadvantaged peers. A relevant point in this report is that most differences in socio-emotional skills were observed within schools, possibly because the development of these skills is often not incorporated into the school curriculum as is the case with the development of cognitive or academic skills (OECD, 2021).

The Collaborative for Academic, Social and Emotional Learning defines SEL as “a process through which all young people and adults acquire and apply the knowledge, skills, and attitudes to develop healthy identities, manage emotions and achieve personal and collective goals, feel and show empathy for others, establish and maintain supportive relationships, and make responsible and caring decisions” (CASEL, 2022). CASEL outlines five competencies associated with SEL skills: self-awareness, self-management, social awareness, relationship skills, and responsible decision making. The CASEL group also makes a case for adopting a developmental lens to foster SEL competencies in childhood, highlighting the need to examine “what changes” and “what remains the same” in children’s SEL skills (Denham, 2018).

The science of school-based SEL has advanced at a steady pace in the past years, and there is now ample evidence to support the inclusion of quality SEL programs in schools (Oberle and Schonert-Reichl, 2017). A large meta-analysis of 213 United States school-based SEL programs (Durlak et al., 2011) suggests that students who participated in such programs improved socio-emotional skills and attitudes, increased prosociality, decreased antisocial behaviors, and accrued gains in academic achievement. A more recent systematic review examined the long-term effectiveness of SEL programs in supporting positive youth development in students (Taylor et al., 2017). The authors reviewed 82 school-based SEL programs offered to children from all grade levels, 38 of which conducted in countries other than the United States. Findings indicated that students who took part in SEL programs in their schools showed continuous gains in terms of social-emotional skills, positive behaviors, and academic achievement, and decreases in emotional distress and behavior problems 4 years after they had completed the programs.

In Portugal, the promotion of social and emotional skills began in the 1990s, with the emergence of intervention programs driven by local entities or associations (Cristóvão et al., 2017). But it was only in 2016 that the Ministry of Health published the *Manual for the Promotion of Social and Emotional Competencies in Schools*. In the year 2021/2022, the National Program for the Promotion of Success in School (*Programa Nacional de Promoção do Sucesso Escolar*) of the Ministry of Education implemented the Plans for Personal, Social, and Community Development in schools (Verdasca, 2022). According to Cristóvão et al. (2017), the first reference to research in social and emotional education in Portugal emerged only in 2011. The authors also claimed that there are few studies regarding SEL implementation in Portuguese schools, and that their relationship with academic success is still quite dispersed.

Still, some studies have revealed the effectiveness of intervention programs to develop children’s social and emotional competence and improve positive behaviors at school (e.g., Moreira et al., 2010; Franco et al., 2017). A 4-year study using a quasi-experimental design evaluated teacher’s interventions in the promotion of social and emotional skills in Portuguese children aged 6 to 10 years, who were attending grades 1 to 4 in primary schools. Teachers taught strategies to children in the experimental group, following a handbook focusing on specific social and emotional skills. After 4 years, significant differences were found for self-control, emotional differentiation, emotional regulation, social skills, and self-esteem (Moreira et al., 2010). In another study with 406 children aged 6 to 11 years in grades 1 to 4, Franco et al. (2017) found social competence to be a mediator of the relationship between emotion understanding and academic achievement facilitating children’s interpersonal interactions.

Altogether, these studies suggest that programs emphasizing socio-emotional learning may promote positive developmental outcomes in students of different ages and from varied backgrounds (see Oberle and Schonert-Reichl, 2017). Arts education programs have long been candidates for the development of socio-emotional skills in children and adolescents. Studies examining social competence are at times confounded with those focusing on socio-emotional development and learning, as seen ahead.

### 1.1. Arts education and children’s social competence: intersections with SEL and current issues

For long have the arts been associated with social life. Thus, it is unsurprising that social development has been associated with arts education. Research on the psychological benefits of arts education on social competence and socioemotional development has expanded considerably in the past few years. While in some studies social and emotional outcomes from participation in different art education modalities are deliberately contrasted (e.g., Archbell et al., 2019), in others, there is a conflation of distinct artistic modalities with outcomes described holistically (e.g., Theodotou, 2017). The latter is more common in studies concerning early childhood.

A main issue with research on arts education and social competence (including SEL) in general, is the treatment of arts education as a “black box,” as if they are a monolithic activity (Ilari, 2020; Holochwost et al., 2021). As Farrington et al. (2019) suggested:

Too often, there is a kind of “black-box” thinking about the connection between arts education and social-emotional learning that obscures, rather than sheds light on, how arts education experiences are frequently described in ways that suggest they have certain ineffable qualities that magically produce social emotional learning in young people (p.12).

Aside from moving away from monolithic views of the arts, it is also important for researchers to delineate the specific domains of socio-emotional development that are being studied, given the complexity of this construct. There is also a need for researchers to outline their theory of change, or “the process that articulates how and why a desired change is expected to happen in a particular context” (Dunphy, 2018, p. 302). The concept of theory of change is rooted in the field of evaluation, and according to Dunphy, was initially motivated by a desire to address causal factors that may lead to change in communities. By addressing theory of change and specificity instead of broad characterizations of both arts and socio-emotional development, we may advance our understanding of the relationships between these two phenomena (Holochwost et al., 2021). Such approach might help us answer important questions. For example, why would a music education program focusing on instrumental performance through written notation develop social awareness in 4th grade children? Similarly, why would an educational program focusing on dramatic storytelling develop prosocial skills in preschool-aged children? What are the characteristics of these programs that make them candidates for transfer into social and emotional skills? Is the researcher’s theory of change logical and plausible?

Specificity is also central to studies on arts education, social competence, and socio-emotional development, as it invites consideration of micro and macro contextual issues, individual/ person factors, and proximal processes, or reciprocal interactions between an individual and one or more persons, objects, or symbols in his/her/their immediate environment (Bronfenbrenner, 2001; Bronfenbrenner and Morris, 2006; Xia et al., 2020). It comes as no surprise then that outcomes in children’s social and emotional areas associated with participation in arts education programs are also contingent upon the nature and characteristics of individual programs (i.e., quality, type, intensity, and duration of instruction; for a discussion see Bohnert et al., 2010). As Holochwost et al. (2021) argued, arts education programs may foster socio-emotional learning in certain domains, but such effects may be more visible in certain populations like younger students. In the next section, we review studies on the two most studied forms of performing arts education in childhood, namely, music and drama with a focus on social competence. The many overlaps between social competence and socio-emotional development are reflected in the existing literature, with studies often concentrating on these different, yet related constructs. It should be noted that our focus is primarily on experimental research conducted in school contexts, as these are the most relevant to the current study.

### 1.2. Music education and children’s social competence

Research on music education and children’s social competence is partly linked to recent theorizing in fields such as musicology, community music, and developmental science, and centers on the associations between musical experiences and social interactions. Much scholarship in music is also predicated on the notion that musical engagement is of a communicative and social nature (see Ilari, 2016). In a seminal text, Small (1998) coined the term “musicking” to describe music as a form of meaningful, collective action that affords humans with opportunities to empathize. This important concept has informed music research from multiple orientations. Scholars in community music often use social learning theories such as legitimate peripheral participation (Lave and Wenger, 1991), and communities of musical practice (Kenny, 2016), to examine collective musical experiences in different settings, aligning somehow with Small (1998). Likewise, the sociology of music offers windows into the role of music in human socialization (Young and Ilari, 2012), and as a technology of the self (DeNora, 2000). Although there are exceptions, most of these works were generated or informed by qualitative methodologies.

Music psychologists, in turn, have typically conducted experiments to demonstrate links between music and social experiences. Some have studied musicality in early child-caregiver communication and interaction (e.g., Malloch and Trevarthen, 1999). Others have examined the associations between musical experiences and prosocial behaviors in young children (Kirschner and Tomasello, 2010; Cirelli et al., 2014). These works suggest that active music participation through performing, moving, singing, and listening may be linked to the different “building blocks” of social competence in early childhood. On a more theoretical level, Koelsch (2013) described seven distinct social functions of music: contact, social cognition, co-pathy (or cognitive empathy), communication, coordination, cooperation, and social cohesion. Cross (2007) argued that collective musical experiences offer humans opportunities to entrain their bodies and voices to a common beat. When musicking with others, a sense of “in-betweenness” may emerge, generating a sense of “we-ness” that is prone to social bonding and cohesion. Rabinowitch et al. (2013) further expanded on this idea and suggested a continuum of intersubjective experiences through music, with a fragmented individual subjectivity on one extreme and a highly coordinated interpenetrating group intersubjectivity on the other extreme. According to them, the latter represents the highest level of understanding of and identification with the other. These ideas seem intuitive and point to the central role of music in the development of social and emotional competence, prosociality and empathy in childhood. Longitudinal studies are an ideal way to test these ideas. Evidence from longitudinal work, however, is still scant and mixed, with only a small number of studies focusing on children’s experiences, particularly in middle childhood.

Some studies found no effects of music instruction on children’s social skills. Schellenberg (2004) assigned 6-year-olds to 4 groups: keyboard, Kodály singing, drama and no lessons (passive control). Children were evaluated on their socio-emotional skills through a parenting rating scale that measures maladaptive and adaptive social functioning before they started learning music and drama in their respective programs and 1 year later. Results showed improvements in adaptive social behavior for the drama group at the post-test, but not for the music group. Rickard et al. (2013) used the teacher form of the Social Skills Rating System (SSRS) to measure the development of social skills in six groups of Australian children over the course of 2 years. Children were grouped based on grade level at the beginning of the study (first or third grades) and activity (music instruction, juggling, and passive controls), and tested 3 times (at baseline, and after 1 and 2 years). Results suggested that there were no effects of either type of training on any of the studied groups.

Other studies have found some associations and effects of music education on children’s social skills. Welch et al. (2014) examined singing and children’s sense of being included in a large sample of British children. They found a positive, linear relationship between children’s mean scores for social inclusion and normalized singing scores. Ilari et al. (2019) examined parental responses to a scale of children’s socio-emotional skills in three groups of children from an underserved community in Los Angeles: music, sports, and control. Children in the music group were attending a collective music education program centering on the performance of orchestral instruments and collective learning through notated music. Children in the sports groups participated in group swim teams and soccer programs, and control children were not attending intensive extracurricular programs. Parents completed the scales before children began their participation in the programs and 4 years later, when children were 11–12 years old. While there were no differences between the groups at baseline, children who participated in music and sports were rated lower in hyperactivity and aggression than controls after 4 years of participation. Also using a longitudinal design, Schellenberg et al. (2015) studied the effects of a mandatory, school-based ukulele program on 3rd and 4th grade Canadian children’s sympathy and prosocial skills. Children who participated in the music program showed higher scores for sympathy and prosocial skills after 10 months of instruction than peers who were not participating in the school music program. However, these effects were limited to child musicians who started off with low scores.

Because the studies reviewed earlier adopted different designs and measures, with testing taking place at different time points and much diversity in program offerings, it is difficult to compare their findings and, consequently, to arrive at a definite conclusion. We interpret this difficulty in terms of a need to conduct more studies on the role of music education on children’s social competence, including replications. As seen ahead, the same can be said in terms of drama education and children’s social competence.

### 1.3. Drama education and children’s social competence

Studies on drama (or theater) education and children’s social competence are partly linked to young children’s engagement in dramatic play (Robertson et al., 2018) and the work of actors, which involves “subtle aspects of their character’s intentions, desires, motivations, beliefs, and emotions, in order to create a realistic portrayal of a complex human onstage or screen (Goldstein and Winner, 2008, p. 230). Although there are monologs, theater is most frequently a collective, performance endeavor, with actors working closely in the construction of characters and interpretation of stories. But why would drama education be a candidate for transfer of learning into social skills?

Cahill (2014) suggested four aspects of drama that may be conducive to the development of social skills: description (i.e., drama to help students explore the workings of the social world and its many complexities); experiential learning (i.e., building self and social awareness, and capacity for imagination and empathy); rehearsal for life (i.e., building communicative skills and problem solving); and critical thinking. Drama education also focuses considerably on role play, inviting children to work with and through their feelings and emotions, learn about others’ perspectives, confront social problems, and affirm identities (Mavroudis and Bournelli, 2016). Güven and Adigüzel (2014) suggested that drama education may also assist students in showing interest in others, giving and receiving, asserting one’s needs and rights in appropriate ways, and being considerate of and sympathetic to others (Güven and Adigüzel, 2014). Neelands (2009) added that drama classes may be a powerful, integrative force to engage students, helping them forge authentic connections between the school curriculum and their own experiences. This occurs, in part, because drama in schools may act as pro-social, ensemble-based processes for building a shared community and culture. Neelands (2009) further stressed that the processes of social and artistic engagement afforded through dramatic experiences are much more important in school contexts than any measurable outcome. As it occurs with music instruction, most studies on learning transfer between drama education and children’s social development is experimental or quasi-experimental in nature, with many adopting longitudinal designs.

Experimental work with preschool-aged children suggests that learning transfer occurs from drama education to children’s social skills. Remziye et al. (2019) examined the effects of 8 weeks of drama education on the social skills of 5-year-olds, who were randomly assigned to experimental and control groups in a Turkish school. The experimental group underwent a drama education program that focused on trust development, emotion recognition, empathic behavior, positive thinking, and communication through dramatic activities, while control children just followed their usual school routine. Using a pre-and post-test design, Remziye and colleagues found children who participated in drama education to receive higher ratings by their teachers on a social skills assessment scale and in all of its subscales (i.e., beginner skills, academic support, friendship, and emotion management). These effects remained for at least another 4 weeks, when preschool children were retested on the same measure. In contrast, Freeman et al. (2010) tested the effects of creative drama on social skills of United States children in grades 3 and 4. Child participants were randomly assigned to four groups, using a Solomon four-group design, and tested on before and after 18 weeks of programming. Children were tested on scales of social concept and the Social Skills Rating System (Gresham and Elliott, 1990). No significant group differences were found for any of the measures.

Concerning other-related social skills, some studies have focused on the effects of drama education on perspective taking, and theory of mind. Goldstein and Winner (2008) studied the associations between drama education and school-aged children’s empathic responding and theory of mind. Children aged 7–11 years were randomly assigned to a drama or visual arts (control) after-school program, consisting of one weekly meeting of 90 min. A pre- and post-test comparison showed a significant effect of drama education on children’s empathy scores. Celume et al. (2020) investigated the potential effects of a drama-based pedagogy centered on social emotional learning on French children’s theory of mind and collaborative skills. Children aged 9 and 10 years from multiple schools were randomly assigned to a 6-week drama program or a control group. A pre-test and post-test comparison revealed effects of the drama program on both theory of mind and collaborative skills.

As with the music education studies, the works reviewed in this section on the impact of drama education on children’s social skills adopted different designs and methodologies, which may partially explain their contrasting results. More research is clearly needed, including in certain areas of the world, like Portugal, where studies on the potential effects of drama education are not common.

### 1.4. Research in arts education in Portuguese schools: connections with social competence

At the time of writing, only a handful of studies were found relating arts education (i.e., performing arts and visual arts) to Portuguese children’s social competence. Studies on the effects of arts education on school-aged children from low-income communities were equally scarce. A small number of studies have focused on the effects of music education in other areas of child development such as motor abilities (Martins et al., 2018). Neves et al. (2021) found associations between vocal emotion recognition and socio-emotional adjustment in 6- to 8-year-old children. In this study, higher emotional prosody recognition was found to be related to the socio-emotional dimensions of prosocial behavior, cognitive and behavioral self-regulation. Neto et al. (2019) examined the impact of a school music education program on Portuguese children’s attitudes toward others, racial and national prejudice. Students who were exposed to a curriculum of Cape Verdean songs during 6 months reduced their prejudice toward Cape Verdean people. Another study (Soares and Lucena, 2013) examined the effects of a one-year dance program on socioemotional skills (e.g., autonomy, acceptance of criticism and respect for others’ space) of low-income Portuguese children aged 6 to 11 years. The program consisted of warm-up, fun activities, and small choreographies with techniques like Hip Hop kids, Modern jazz and traditional Portuguese and international dances. Findings suggested that participation in the program improved children’s acquisition of rules, acceptance of criticism, autonomy, and cooperation (Soares and Lucena, 2013).

Throughout this review, it becomes evident that more research is needed to examine the potential benefits of arts education on children’s social competence, in Portugal and elsewhere. As Holochwost et al. (2021) have contended, it is important that researchers define the social skills of interest and detail the arts programs that are being studied and present their theory of change (Dunphy, 2018). Researchers should also consider the inclusion of populations that have not been represented in the literature and replicate earlier studies by adopting designs, scales and instruments that have already been used. Therefore, in this study, we focused purposefully on the development of social skills, or the building blocks of children’s social competence, which are malleable in childhood (Little et al., 2017). A second reason to focus on social skills was the fact that they have been thoroughly examined, with measures developed and validated for Portuguese samples like the SRSS (Lemos and Meneses, 2002), allowing for comparisons with earlier studies (e.g., Rickard et al., 2013). In this study, we adopted Gresham and Elliott’s (1990) definition of social skills, or behaviors that benefit others and improve social interactions. The components examined in the current investigation were social skills, behavioral problems and academic competence (Gresham and Elliott, 1990), as described ahead.

### 1.5. Study aim

The aim of this study was to examine the role of music and drama education programs on the development of social skills of Portuguese school-aged children from disadvantaged communities over the course of one school year. We hypothesized that carefully-designed music and drama programs, with performing, creating, and listening activities, delivered collectively during regular school hours, and led by specialist, highly committed teachers/performers, could motivate and engage children in the learning process, having an impact on their social skills.

## 2. Materials and methods

### 2.1. Participants

Portuguese children (*N* = 169) with a mean age of 6.84 years (range = 6–8 years) attending the second year of primary education in public schools were recruited for this study. From the initial 169 children, 10 moved to other schools in the middle of the year (AR = 5.91). The final sample consisted of 159 children (80 boys; *M_age T0_* = 6.84; *SD* = 0.43; *M_age T1_* = 7.57; *SD* = 0.52). Most children (*n* = 110, 69%) came from families living with a yearly income lower than 9.215 euros, an amount way below the average salary of 23,200 euros for the year of 2021 in Portugal (OECD, 2022). These families were eligible for social programs like the “School Social Action” (*Ação Social Escolar*), which provides disadvantaged children with free daily meals and school supplies.

All participating children came from 10 classrooms in eight public schools that were located in deprived areas, in the city of Porto. Participating classes were selected based on two criteria: belonging to (1) schools identified by the Portuguese government as Educative Territories of Priority Intervention (*Territórios Educativos de Intervenção Prioritária*—TEIP; OECD, 2014), which are schools located in areas with economic and social problems; and (2) schools that did not offer music education as curricular or extracurricular activities. The mean classroom size was 16 students (range 12–20). The 10 classrooms were randomly assigned to one of three experimental conditions: (1) Music (*n* = 51), children involved in a music program during the school year; (2) Drama (*n* = 56), children involved in a drama program during the school year; (3) and Waitlist-Control (*n* = 52), children who were not attending any school-based, Music and Drama activities during the school year. For ethical reasons, children in the control group were offered music in the subsequent year following data collection.

At the beginning of the study, all 10 classroom teachers had been teaching their classes for one school year (1st grade). All teachers were females (ages 38–62 years) and had 15 to 34 years of teaching experience. All teachers obtained teaching degrees in Primary Education; Four teachers obtained a post-Graduate certificate (Special Education, Arts), and two had master’s degrees (Educational Supervision, Psychology). Regarding their musical experience, two teachers had instrumental lessons as a child, but most reported no formal music training.

Study protocols followed the Porto Polytechnic Code of Practice. The study was fully explained to the children, legal guardians, classroom teachers and school principals before consent or approvals were obtained. Protocols were signed between each school principal and the CIPEM/INET-md research center, which was registered at the Portuguese Ministry of Education. All ethical procedures were followed, and parents and teachers signed informed consent forms before any data were collected.

### 2.2. Music education and drama education programs

Music and drama were selected as our main areas of study for the following reasons. First, because they are two types of performing arts that have been linked to children’s daily experiences through acts of play (Cahill, 2014). Second, elements of music and drama are commonly infused in primary education, and this is true even for schools that do not offer specialized instruction in these areas. Primary school teachers often make use of songs, skits, and dramatic elements such as storytelling to teach and illustrate concepts and ideas. Thus, the incorporation of programs focusing on music and drama would not be completely foreign to the school community. Third, music and drama are art forms that tap into emotions, as they rely on external representations of individual and collective views of the world (Cahill, 2014). Through musical and dramatic work, children learn to recognize the emotions of composers, artists, audience members and their own. Drama education, in particular, deals with the representation of others, which is directly linked to theory of mind and empathy (Goldstein and Winner, 2008). Music education, in turn, requires children to develop a wide range of skills, including rhythmic entrainment capacities that allow them to perform, play, sing or move with others. Earlier research suggests that even short, collective and synchronized musical experiences in childhood may lead children to be more prosocial with their peers (Kirschner and Tomasello, 2010). Both music and drama education in primary school are usually taught in groups, and children are encouraged to listen to each other, share experiences, take turns, and build a sense of community through formal and informal performances (Neelands, 2009). Music and drama programs are also designed to engage and motivate children, and have the potential to enhance children’s attention, memorization, communication, cooperation, and emotional expression (see Goldstein et al., 2017). By practicing these skills through a yearlong music or drama program, we anticipated that children would potentially develop cooperation and self-control, reduce problem behaviors, and enhance academic competence (see Gresham and Elliott, 1990).

The active music education program (hereinafter *music program*) was implemented during regular school hours, in the morning period. Children attended weekly classes for 60 minutes each over the course of 30 weeks, or one school year. The program was delivered by a specialist music teacher, and was based on active and participatory music pedagogies, consisting of listening, performing, singing, playing, movement, dancing, and creating music (Wuytack and Boal-Palheiros, 2013; Boal-Palheiros, 2015). Musical activities included listening to music from different cultures and styles; practicing vocal exercises and breathing; playing musical games; learning technical and expressive aspects of musical performance; learning and performing songs; playing instruments such as small percussion; using spoken and sung voice, body, and objects as musical instruments; creating music by exploring different sounds and musical elements; improvising rhythms and melodies; moving and dancing; taking part in short concerts performed for their families; evaluating their musical performances. The musical repertoire comprised various genres and styles, including children’s preferred ones (e.g., classical, contemporary, pop, rock, and traditional). The teaching strategies demanded children’s active engagement with music through listening, modeling, collective singing and playing, improvising, composing, performing with and for others, and talking about musical activities. Technological resources were used to enhance interactive learning.

The dramatic expression program (hereinafter *drama program*) was also implemented during regular school hours, in the morning period. Children in the drama group attended weekly classes of 60 minutes each for 30 consecutive weeks. The program was delivered by a specialist drama teacher, and was also based on active and participatory pedagogies, consisting of group creative, performance and movement activities (Goldstein et al., 2017). Like music, drama is an artistic performing art with an auditory component, involving practice and rehearsal, memorization, learning new scripts or pieces, and expressing emotions. Drama activities included improvising and role-playing; dramatic games; group dynamics, breathing and movement exercises, learning and practicing observation skills (e.g., observing others and being observed); communicating and interacting with peers; exploring expressive possibilities of voice and body, and the collective space; creating and dramatizing stories; learning, memorizing, and performing skits; taking part in performances for their families; evaluating their performances. The teaching strategies also demanded children’s active engagement with the drama activities, all of which were carried out in groups. The children in the control group followed their normal school routine and carried out regular curricular activities with their classroom teacher.

In each program, teaching quality was ensured through the selection of music and drama teachers with solid pedagogical skills, long experience teaching children, and personal qualities, such as the ability to establish positive interactions with students. The weekly-lesson plans and teaching strategies were carefully elaborated and regularly monitored by a researcher experienced in teacher supervision. In both programs, the classroom teachers attended the lessons, and some lessons were observed. Both the classroom and the specialist teachers reported a high degree of children’s engagement in the music and the drama activities, which reinforced our confidence in the potential of both art programs to improve children’s outcomes (Wiens and Gordon, 2018).

Table 1 depicts the objectives of both Music and Drama programs, which were based on the Portuguese guidelines for Music and Drama in Primary education (DGE—Direção-Geral de Educação, 2001, 2018a,b). The activities carried out were planned by the specialist teachers and the authors. We also provide examples of activities that could potentially promote the development of social skills.

**Table 1:** Music and drama: objectives and activities promoting children’s social skills.

Music objectives	Musical activities	Promoting social skills
Develop listening skillsDevelop children’s musical culturesDevelop music performance skills, using voice, body, and instrumentsDevelop skills to improvise musicDevelop skills to create musicDevelop skills to move and dancePromote musical enjoymentDevelop children’s socio-emotional skills – self-esteem, self-confidence, sense of belonging, through active participation in personal and collective challenges	Listening to different soundsListening to and appreciating music from various styles and culturesSinging songsPlaying musical instrumentsUsing spoken and sung voice, body, and objects as musical instrumentsImprovising rhythms and melodiesCreating music by exploring different musical elementsMoving and dancing to the musicEvaluating their musical performancesKnowing and discussing about music	Listen to other’s musicListen to others’ musical partsSing together in tuneSing synchronouslyPlay music synchronouslyMove synchronously to musicExpress, discuss and negotiate musical ideas in the groupCommunicate with peers through music and movementShare musical experiencesRespect the rulesIntegrate in the groupInteract and cooperate with peers
Drama objectives	Dramatic activities	Promoting social skills
Develop bodily, vocal, and verbal expressionDevelop awareness of the communicative bodyDevelop spatial awarenessDevelop observation skillsDevelop creative thinkingDevelop skills to improvise and actDevelop respect for the collective spaceDevelop esthetic and critical skillsDevelop children’s socio-emotional skills	PlayingImprovisingRole-playingPerformanceDramatic gamesGroup dynamicsExercises of breath, movement, and improvisationObserving others and being observed by othersAdapting expressive possibilities of the voice to different situationsPresenting performances to othersEvaluating their performances	Express ideas verballyListen to others’ opinions and storiesListen to and negotiate with othersUnderstand and accept differencesFeel the silenceWalk in the space and find a common tempoCommunicate and interact with others, through the voice and the bodyShare performance activities with the groupRespect the rulesIntegrate in the groupInteract and cooperate with peers

### 2.3. Study design

This study is part of a larger, longitudinal study that examined potential effects of music and drama education on children’s cognitive, emotional, musical, academic, and social development. In this paper, we report data on the development of social skills in three groups: music, drama, and passive control, over the course of 1 year, using a pre-test and post-test design. Children’s musical skills, including singing, rhythmic and melodic performances are discussed elsewhere.

### 2.4. Measures and procedures

The Social Skills Rating System or SSRS (Gresham and Elliott, 1990), a widely used measure of children’s social behaviors, has been used in schools across many countries (Gresham et al., 2011). In this study, we used the SSRS—Teacher Form, adapted for the Portuguese population by Lemos and Meneses (2002). Teachers provide useful information about children’s behavior and academic competence, through observed behaviors over long periods of time (Lemos and Meneses, 2002). This instrument requires teachers to rate their students’ social skills over 54 items on a 3-point frequency scale (1 = Never, 2 = Sometimes, 3 = Very Often). The SSRS Portuguese adaptation includes three social skills domains. Cooperation (10 items) refers to behaviors related to sharing and following the rules (e.g., “performs tasks on time”). Assertion (10 items) assesses how often children take initiatives, and their reactions to others (e.g., “initiates conversations with peers”). Self-Control (10 items) measures children’s reactions in conflict situations (e.g., “reacts adequately when pushed by other children”). The SSRS also includes three problem behavior domains, namely, Externalizing Problems (7 items), Internalizing Problems (6 items) and Hyperactivity (5 items). It also includes a scale of Academic Competence (global, Reading/Portuguese and Mathematics), with 6 items that are rated in a 5-point scale. These different domains yield three scores: social skills, behavior problems, and academic competence. The internal consistency (Cronbach’s Alpha) of these scales is high: 0.93 for Social Skills, 0.91 for Behavior Problems, and 0.96 for Academic Competence (Lemos and Meneses, 2002).

In earlier studies, the SSRS Portuguese adaptation revealed some interesting findings. One study examined the relationship between 8- and 9-year old children’s social competence and their mental representations of parents. Children who perceived their parents as rejecting and punitive figures were rated by their teachers as being less competent in social skills (i.e., cooperation, assertion, self-control), and also had more internalized behavior problems and less academic competence (Custódio and Cruz, 2008). Another study showed a strong association between social competence and academic competence in third-grade children. Children with higher academic competence also had more social skills and less behavior problems than those with lower academic competence (Cardoso and Veríssimo, 2010). Using a cross-sectional design with 360 students from three school grade cohorts (3rd, 5th, and 7th), Freire et al. (2020) examined the associations between student behavior problems, social skills, and academic performance, and teachers’ perceptions of conflict and closeness in relationships with students. Results showed that teachers had a positive perception of student social skills, and also perceived their own relationships with students as positive, reporting relatively high levels of closeness and low levels of conflict (Freire et al., 2020).

In the present study, the researchers asked classroom teachers to complete the SSRS—Teacher Form twice: at the beginning (t0) and at the end of the school year, after the music and drama programs had ended (t1). Data were scored according to the norms found in the SSRS manual.

## 3. Results

Descriptive analysis was conducted for all variables in SSRS dimensions and domains per testing time (t0—baseline and t1—post-test see Table 2). In order to understand the impact of music and drama education programs on children’ social skills, we performed a Mixed ANOVA for each of SSRS social skills domains (i.e., cooperation, assertion, self-control), behavioral problems domains (i.e., externalizing behaviors, internalizing behaviors, hyperactivity) and academic competence—considering group (M—Music, D—Drama, C—Control) as the between-subjects factor, and testing time (t0, t1) as the within-subjects factor. Mixed ANOVAS were also performed with two composite variables: social skills and behavioral problems. In case there were interaction effects between conditions, additional mixed ANOVAS were conducted between two group conditions (M-D, M-C, and D-C). Effect sizes are reported for all significant effects, using multivariate partial eta squared. Eta-squared values of 0.01, 0.06, and 0.14 are interpreted as small, medium, and large effects, respectively (Cohen, 1988).

**Table 2:** SSRS descriptive statistics per group.

	Music (*N* = 51)		Drama (*N* = 56)		Control (*N* = 52)	
	*t0*	*t1*	*t0*	*t1*	*t0*	*t1*
	*Mean (SD)*	*Mean (SD)*	*Mean (SD)*	*Mean (SD)*	*Mean (SD)*	*Mean (SD)*
Cooperation	23.24 (4.03)	23.02 (3.87)	22.11 (4.39)	23.21 (4.25)	25.27 (3.89)	24.90 (4.22)
Assertion	22.18 (4.18)	23.33 (4.68)	21.18 (3.61)	23.27 (4.08)	25.02 (3.98)	23.21 (4.14)
Self-control	23.25 (3.56)	24.24 (3.77)	21.89 (3.74)	23.66 (4.05)	24.65 (3.90)	23.33 (4.25)
Social skills	68.67 (10.30)	70.59 (10.82)	65.18 (10.34)	70.14 (10.80)	74.94 (10.14)	71.44 (10.85)
Externalizing problems	10.20 (2.93)	9.88 (2.59)	10.38 (3.25)	10.10 (3.43)	10.46 (3.72)	11.02 (3.17)
Internalizing problems	8.69 (1.68)	8.25 (1.62)	8.70 (2.30)	7.96 (2.00)	10.10 (2.23)	11.02 (2.80)
Hyperactivity	9.00 (2.24)	8.55 (1.85)	10.02 (2.77)	9.23 (2.55)	9.35 (2.34)	9.17 (2.59)
Behavioral problems	27.88 (5.66)	26.69 (4.11)	29.09 (6.92)	27.20 (6.64)	29.90 (6.20)	31.21 (6.43)
Academic competence	17.94 (5.46)	18.55 (4.74)	17.70 (5.50)	18.41 (5.89)	18.48 (4.71)	20.02 (5.86)

### 3.1. Social skills: cooperation

**Figure 1:**
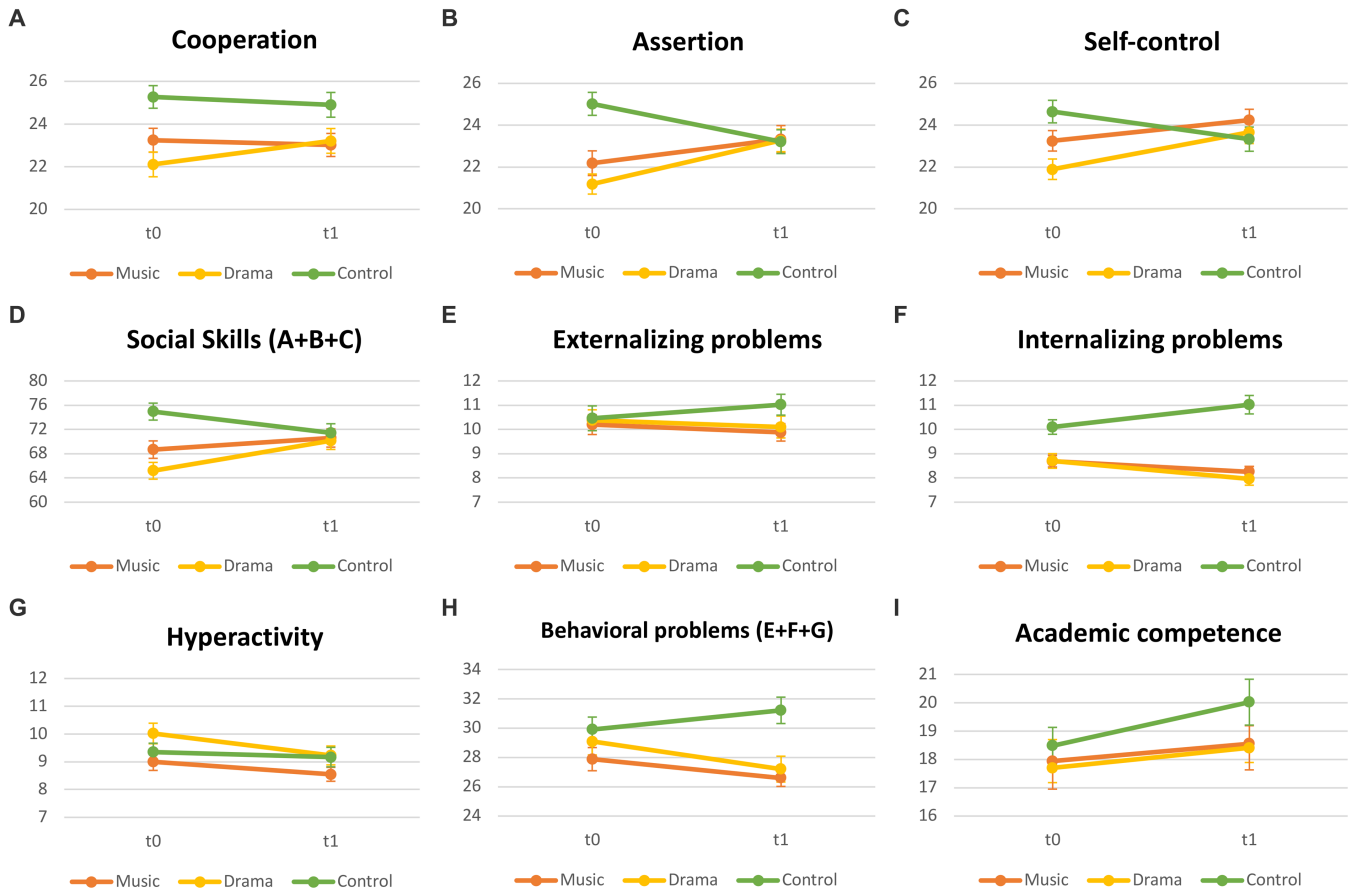
SSRS domains at t0 and t1. Error bars represent SEM. Axis x represents the two data collection times. Axis y represents the mean scores on each SSRS domain.

A mixed ANOVA was conducted to analyze the effect of time and group condition on the cooperation domain (Figure 1A). There was a significant and moderate size interaction effect between time and group condition [*F*(2, 156) = 5.85, *p* < 0.01, *η*^2^_p_ = 0.07]. Simple main effects analysis showed no significant time effect on cooperation domain [*F*(2, 156) = 0.798, *p* = 0.37, *η*^2^_p_ = 0.005] but a moderate significant effect of group condition [*F*(2, 156) = 5.69, *p* < 0.01, *η*^2^_p_ = 0.07] on cooperation scores. Additional analyses show significant, moderate interaction effects between M and D [*F*(1, 105) = 7.15, *p* < 0.01, *η*^2^_p_ = 0.07], and between D and C conditions [*F*(1, 106) = 9.07, *p* < 0.01, *η*^2^_p_ = 0.08], but no significant differences on interaction effects between M and C [*F*(1, 101) = 0.11, *p* = 0.74] on this domain. Thus, cooperation scores increased in the Drama group between the pre-test and the post-test, while there was a slight decrease in the Music and the Control groups (Table 2; Figure 1A).

### 3.2. Social skills: assertion

Simple main effects analysis showed no significant time effect on this domain [*F*(2, 156) = 3.55, *p* = 0.06] but a significant, although small effect of group condition [*F*(2, 156) = 3.53, *p* < 0.05, *η*^2^_p_ = 0.04] on assertion scores (Figure 1B). There was a significant and large size interaction effect between time and group condition [*F*(2, 156) = 21.42, *p* < 0.001, *η*^2^*_p_* = 0.22]. Additional analyses showed no significant interaction effects between M and D [*F*(1, 105) = 2.13, *p* = 0.15], but significant large effects between D and C conditions [*F*(1, 106) = 41.08, *p* < 0.001, *η*^2^_p_ = 0.28] and between M and C conditions [*F*(1, 101) = 22.65., *p* < 0.001, *η*^2^_p_ = 0.18]. Assertion scores increased for both the Music and the Drama groups from pre- to post-test and decreased in the Control group (Table 2; Figure 1B).

### 3.3. Social skills: self-control

Simple main effects analysis showed that neither time [*F*(1, 156) = 3.72, *p* = 0.056] nor group condition [*F*(2, 156) = 1.76, *p* = 0.175] had significant effects on self-control scores (Figure 1C). These results could be due to crossover effects. Nevertheless, there was a significant interaction between time and group condition [*F*(2, 156) = 14.37, *p* < 0.001, *η*^2^_p_ = 0.16], and the effect size was large. Additional analyses showed no significant interaction effects between M and D conditions [*F*(1, 105) = 1.50, *p* = 0.22], but significant and large differences between D and C [*F*(1, 106) = 28.47, *p* < 0.001, *η*^2^_p_ = 0.21] and between M and C [*F*(1, 101) = 16.02, *p* < 0.001, *η*^2^_p_ = 0.14]. Self-control scores increased for both the Music and the Drama groups between the pre- and post-test and decreased for the Control group (Table 2; Figure 1C).

### 3.4. Social skills (composite)

A composite variable (sum of scores on cooperation, assertion, and self-control) was created for inspecting the social skills dimension (Figure 1D). Considering main effects, a marginal and small effect was found for time [*F*(2, 156) = 3.88, *p* = 0.051, *η*^2^_p_ = 0.02], and also for group conditions [*F*(2, 156) = 4.49, *p* < 0.05., *η*^2^_p_ = 0.05]. A mixed ANOVA was conducted on social skills scores revealing an interaction and large effect between time and group conditions [*F*(2, 156) = 18.89, *p* < 0.001, *η*^2^_p_ = 0.20]. Significant and large interaction effects sizes were found between D [*F*(1, 106) = 40.40, *p* < 0.001, *η*^2^_p_ = 0.28] and M [*F*(1, 101) = 15.40, *p* < 0.001, *η*^2^_p_ = 0.13] when compared to C. A significant and small effect was also found between D and M [*F*(1, 105) = 4.17, *p* < 0.05, *η*^2^_p_ = 0.038]. Children who participated in the Music and the Drama groups were rated higher in social skills (composite) by their teachers in the post-test (Table 2; Figure 1D).

### 3.5. Behavioral problems: externalizing problems

There was no significant interaction effect between time and group condition [*F*(2, 156) = 2.23, *p* = 0.11], neither significant main effects for time [*F*(2, 156) = 0.047, *p* = 0.83] and for group conditions [*F*(2, 156) = 0.082, *p* = 0.044] on externalizing problems scores (Table 2; Figure 1E). There were no significant group differences in terms of ratings for externalizing problems following 1 year of music and drama programs.

### 3.6. Behavioral problems: internalizing problems

Simple main effects analyses showed no significant time effect on this domain [*F*(2, 156) = 0.30, *p* = 0.58], but there was a significant and large effect of group condition [*F*(2, 156) = 21.75, *p* < 0.001, *η*^2^_p_ = 0.22] on Internalizing Problems scores (Figure 1F). There was a significant and large size interaction effect between time and group condition [*F*(2, 156) = 12.15, *p* < 0.001, *η*^2^_p_ = 0.14]. Significant interaction effects sizes were found between both D [*F*(1, 106) = 23.97, *p* < 0.001, *η*^2^_p_ = 0.19] and M [*F*(1, 101) = 13.55, *p* < 0.001, *η*^2^_p_ = 0.12] when compared to C, but no significant effects were found between D and M conditions [*F*(1, 105) = 0.67, *p* = 0.42, *η*^2^_p_ = 0.01]. There was a decrease in ratings for internalizing problems for the Music and the Drama groups, and an increase for children in the Control group (Table 2; Figure 1F).

### 3.7. Hyperactivity

Simple main effects were only significant for time [*F*(2, 156) = 10.29, *p* < 0.01] but not for group conditions [*F*(2, 156) = 1.94, *p* = 0.15; Table 2; Figure 1G]. There was no significant interaction effect found between time and group condition [*F*(2, 156) = 0.1449, *p* = 0.23]. There were no significant differences between the three groups for hyperactivity following 1 year of music and drama education.

### 3.8. Behavioral problems (composite)

Following the SRSS manual, a composite variable for behavioral problems was also created (sum of scores on externalizing problems, internalizing problems, and hyperactivity; Figure 1H). A mixed ANOVA was performed and revealed a significant and moderate effect of group [*F*(2, 156) = 4.77, *p* < 0.05., *η*^2^_p_ = 0.06], but no effect for time [*F*(2, 156) = 2.43, *p* = 0.121]. There was a significant and moderate interaction effect between time and group conditions [*F*(2, 156) = 6.53, *p* < 0.05, *η*^2^_p_ = 0.08]. Significant interaction effects and moderate effects sizes were found between D [*F*(1, 106) = 12.51, *p* < 0.001, *η*^2^_p_ = 0.11] and M [*F*(1, 101) = 7.70, *p* < 0.01, *η*^2^_p_ = 0.07] when compared to C. No effect was found between D and M [*F*(1, 105) = 4.97, *p* = 0.48]. Children’s behavioral problems were reported to decrease for both the Music and the Drama groups following 1 year of programming, whereas there was an increase in behavioral problems for the Control group (Table 2; Figure 1H).

### 3.9. Academic competence

A Mixed ANOVA revealed a main effect of time on this domain [*F*(2, 156) = 13.12, *p* < 0.001, *η*^2^_p_ = 0.08], but no significant effect for group condition [*F*(2, 156) = 0.83, *p* = 0.44]. There was no significant interaction effect between time and group condition [*F*(2, 156) = 1.23, *p* = 0.30] (Figure 1I). Academic competence improved for all children; there were no significant group differences for this variable (Table 2; Figure 1I).

## 4. Discussion

The aim of this study was to examine whether music or drama education in primary schools could contribute to the development of social competence of Portuguese children from disadvantaged communities. Most participating children belonged to low-income families and did not have access to music or drama at school, either as curricular or extracurricular activities. We anticipated that the carefully designed music and drama programs, delivered in schools by specialist and highly motivated teachers, would have an impact on children’s social competence. We assessed children’s social competence by examining three domains: social skills, behavior problems, and academic competence using the SSRS—Teacher Form.

In longitudinal research with children, it is typical for effects of time (also known as developmental effects) to surface (see Ilari, 2020). Developmental effects were found for academic competence and hyperactivity, and marginal effects were found for social skills (composite) scores. In other words, children in our study received higher ratings for academic competence and lower ratings for hyperactivity at the post-test. These effects were likely linked to children’s development and growth. Considering overall social skills, it was noteworthy that children in the music and the drama groups were generally rated lower in social skills (composite) than controls at the pre-test, whereas both groups were rated higher at the post-test. These results are in line with studies that used the same measurement and found effects of drama (Remziye et al., 2019), and with another study that suggested effects of music education on children’s social skills, but only in those who started off with low scores (Schellenberg et al., 2015). Our results also contradict findings from previous studies that used the SRSS measurement and found no effects of arts education on social skills (e.g., Freeman et al., 2010; Rickard et al., 2013). These contrasting findings may be due to differences in curricula in the music and drama programs.

In terms of the SRSS subscale, some interesting findings emerged. Children in the control group received higher ratings for cooperation than children in the music and drama groups at the pre-test. Yet, the drama group showed more improvement in their ratings for cooperation at the post-test, which did not occur with the music and control groups. This finding is consistent with earlier research that found improvements in adaptive social behavior for children involved in drama programs (Schellenberg, 2004; Celume et al., 2020). We speculate that the drama group showed more improvement in ratings at the post-test due to the collective and cooperative nature of the drama education. As noted earlier, drama education involves a huge amount of dialog and group decision making, and perhaps more so than music. Music education, in turn, requires repetition and focus on mastering individual skills (e.g., matching pitches, playing rhythms, and synchronizing) within a group setting. Thus, it is possible that cooperation in music programs may depend, to some degree on the mastering of musical skills. In music programs, it is possible that children may need to master individual skills first, before they are ready to engage in cooperative tasks, like performing or creating music together. These are speculations that need to be further examined.

Interestingly, children in the control group received higher ratings for assertion than their music and drama counterparts in the pre-test. But at the post-test, the control group was rated lower, while both music and drama groups were rated higher by their teachers. This finding suggests that children developed their capacity to initiate social interactions and express their views (see Gresham, 2001), and is also consistent with the nature of music and drama education programs, which involve dialog, collective singing, acting, and dancing. Similarly, our data showed that children in music and drama received higher ratings for self-control at the post-test, with the control group receiving lower ratings from their classroom teachers. In both music and drama classes, children learn to listen to one another and to take turns, which are key for the development of self-control. This finding lends support to earlier research that suggested an impact of arts education in music (Winsler et al., 2011; Hennessy et al., 2019), and drama (Çiftçi and Aykaç, 2022) on children’s executive function skills.

Two other findings are worthy of commentary. First, it was interesting how control children received higher ratings in general. This finding is likely related to school and teacher variables. Second, while the ratings for music and drama groups for social skills, assertion, and self-control increased from the pre-test to the post-test, they decreased for the control group. Similarly, ratings for controls increased for internalizing and behavioral problems, yet decreased for the music and drama groups for these skills. These findings are aligned with an earlier study that found primary school children to receive lower ratings for aggression following participation in formal music education (Ilari et al., 2019). These results also suggest that, at least in the eyes of teachers, children in the music and drama programs developed specific social skills over the course of 1 year. We suspect that drama education may have assisted in the reduction of internalizing and behavioral problems, due to their emphasis on experiential learning and problem solving and work on communicative skills (Cahill, 2014). The yearly music and drama education program likely afforded students with opportunities to listen to self and others, tapping into their prosocial skills (Kirschner and Tomasello, 2010; Güven and Adigüzel, 2014).

While curricular differences may have accounted for differences between our findings and those of other studies, including some that used the same measurement (e.g., Freeman et al., 2010; Rickard et al., 2013) there are other possible explanations. First, our study focused on children from low socioeconomic backgrounds. It is possible that the novelty and quality of the programs may have served as a motivating factor for children to not only engage in the artistic activities, but also develop social skills around them. Earlier research has suggested that student age, the quality of programs (see Holochwost et al., 2021), and the quality of teaching (Hallam, 2015) may have an impact on outcomes. Second, macro-time issues (Bronfenbrenner and Morris, 2006) may have affected teacher responses. Our study took place during the year 2021–2022, when the world was still under the restrictions imposed by the COVID-19 pandemic. It is possible that music and drama programs served as a protective factor, by giving children opportunities to engage in collective activities with their peers that allowed for self-expression and work through emotions. This might also explain why the control group, which started off with higher ratings in the pre-test for most sub-skills, showed a decrease in ratings in the post-test.

Overall, our findings provide support for arts education in schools, suggesting that their implementation during one school year may have improved children’s social skills, such as assertion and self-control in students participating in drama and music, and cooperation in the drama group. We suggest that participation in music and drama programs may also serve as a protective developmental factor, helping children develop artistic competencies, self-regulation, and assertiveness, and potentially reducing internalizing and behavioral problems.

### 4.1. Strengths, limitations and directions for future research

Our study is likely the first to examine the role of music and drama programs on social skills of children from underserved communities in Portugal. We examined children’s experiences as they underwent a carefully designed curriculum implemented by experienced teachers in schools, at a time when attention to student social and emotional skills and mental health are considered paramount (Kauhanen et al., 2022; Sohn, 2022). Because social competence is a complex construct that has been studied in multiple ways, few studies to date have used social skills measures that consider its multidimensionality or have used well-established and validated measures (Sørlie et al., 2021). In this study, we aimed to partially address these problems by utilizing the SRSS measure (Gresham et al., 2011). But like every study, ours had some limitations.

First, our study relied on teachers’ perceptions of frequency of children’s behaviors. But even if some may argue that teachers’ perceptions may suffer from biases, we argue that their views are rather important as children spend many hours at school. Teachers not only observe them daily for long periods of time but have been found to be reliable raters of children’s social skills (Sørlie et al., 2021). In order to get a deeper understanding of the dimensions of children’s social competence, data in the form of parental and children’s self-ratings could be included in future studies, as suggested in the SSRS manual (Gresham and Elliott, 1990). This would allow for a coordinated evaluation from multiple informants in different contexts (school, home) and from different perspectives.

Children’s social functioning and development of social skills are affected by the context in which they develop, such as home, school and communities, along with classroom environment, peer relationships and interactions with teachers (Sørlie et al., 2021). School-related factors such as schedules and routines, might have influenced the development of children’s social competence, yet it was not possible to control for the variables linked to school, including classroom placement and teacher. Future studies could examine these variables in more detail by including qualitative data. Periodic observations, diaries, and interviews with children and families can provide insightful information about children’s participation in school-based music and drama programs and social skills over time.

The length of children’s musical engagement and commitment is also worthy of commentary, given its relevance to learning and development (Hallam, 2015). In our study, the one-year availability of participating schools did not allow for longer interventions. Future studies could consider the duration of the intervention programs, by extending the music and the drama programs for 2 or 3 years, as education is a process that occurs over time. Effects of music and drama programs may be more visible following longer periods. Along similar lines, retesting children a few months or years after the end of the programs would help determine whether effects were long term. Future research could also gauge the views of children and participating teachers about both the music and the drama programs and factor them into data analysis. Whether engagement is a positive experience also contributes to whether personal change is beneficial or not (Hallam, 2015).

In our study we relied on specialist and highly motivated music and drama teachers, which some could view as more of an exception as most primary schools do not have access to specialist art teachers. And although the primary school curriculum in Portugal includes Artistic Education—Visual Arts; Dramatic Expression/Theater; Dance; Music—most classroom teachers do not teach the arts regularly in their classrooms (Boal-Palheiros and Encarnação, 2008; Noble, 2021). Most teachers possess generalist training and often lack specialized artistic training, with many being overly concerned with teaching the core subjects, such as Portuguese and Mathematics. While our study findings support the provision of quality arts education in primary schools, we also recognize the urgent need for increasing arts education in professional development and teacher training programs.

In the current study, we specified objectives and activities of the music and the drama programs (Table 1) and attempted to relate them to the development of specific social skills in school-aged children, following the suggestions of Holochwost et al. (2021). Yet it is not possible to pinpoint the exact activities in the music and drama that were responsible for change in children’s social skills. Education and development are intricate processes. Music and drama, in turn, are complex areas with elements that are difficult to disentangle. We suspect that it was the combination of activities along with the novelty of being in engaging music and drama programs that might have led to changes in teachers’ ratings of students. Thus, a challenge for future research is to investigate which specific aspects of the activities carried out in music, drama and other art programs may lead to development in specific skills associated with children’s social competence.

## Data availability statement

The raw data supporting the conclusions of this article will be made available by the authors, without undue reservation.

## Ethics statement

This study follows the Porto Polytechnic Code of Practice. A Declaration of Commitment was issued by the Porto Polytechnic. Written informed consent to participate in this study was provided by the participants’ legal guardian/next of kin. Children also assented to participate.

## Author contributions

GB-P designed the study and collected the data. GB-P and BI analyzed and interpreted the data and wrote the manuscript. All authors contributed to the article and approved the submitted version.

## Funding

This study was financed by the Foundation for Science and Technology (Fundação para a Ciência e a Tecnologia), projects UIDB/00472/2020 and UIDP/00472/2020, Portugal.

## Conflict of interest

The authors declare that the research was conducted in the absence of any commercial or financial relationships that could be construed as a potential conflict of interest.

The handling editor GFW declared a past co-authorship with the author BI.

## Publisher’s note

All claims expressed in this article are solely those of the authors and do not necessarily represent those of their affiliated organizations, or those of the publisher, the editors and the reviewers. Any product that may be evaluated in this article, or claim that may be made by its manufacturer, is not guaranteed or endorsed by the publisher.
